# Urolithin A preserves renal perfusion and function and attenuates inflammasome-associated signaling in a translational porcine model of sepsis-induced acute kidney injury

**DOI:** 10.21203/rs.3.rs-10630708/v1

**Published:** 2026-08-26

**Authors:** Adebowale Adebiyi, Julia de la Cruz, Olugbenga Michael, Praghalathan Kanthakumar

**Affiliations:** University of Missouri - Columbia; University of Missouri - Columbia; University of Missouri - Columbia; University of Missouri - Columbia

**Keywords:** Fecal peritonitis, sepsis, acute kidney injury, urolithin A, porcine model, NLRP3 inflammasome, pyroptotic signaling

## Abstract

Sepsis-associated acute kidney injury (SA-AKI) is a major complication of critical illness for which no targeted therapy is available. Urolithin A (UA), a gut microbiota-derived metabolite with anti-inflammatory and antioxidant properties, has shown organ-protective effects in preclinical studies, but its effects on renal physiology during sepsis remain unknown. We evaluated UA in a translational porcine model of autologous fecal peritonitis-induced sepsis using 6–8-week-old male Landrace × Large White crossbred pigs. Animals were assigned to sham, fecal peritonitis (FP), or FP plus intravenous UA administered after sepsis induction. At 24 hours, systemic and renal hemodynamics, glomerular filtration rate (GFR), kidney injury biomarkers, inflammatory and oxidative stress indices, and renal inflammasome-associated proteins were assessed. Complementary studies used lipopolysaccharide-challenged primary porcine proximal tubular epithelial cells. FP produced a hypodynamic septic phenotype with systemic and renal hypoperfusion, increased renal vascular resistance, decreased GFR, and increased kidney injury biomarkers. UA attenuated hemodynamic abnormalities, preserved GFR, and reduced kidney injury, inflammation, and oxidative stress. FP increased renal immunoreactivity for NLR family pyrin domain containing 3 (NLRP3), apoptosis-associated speck-like protein containing a caspase recruitment domain (ASC), cleaved caspase-1, gasdermin D N-terminal fragment, interleukin-1β, and interleukin-18; these responses were attenuated by UA. In porcine proximal tubular cells, UA reduced LPS-induced caspase-1 activity and expression of NLRP3, cleaved caspase-1, and gasdermin D N-terminal fragment. These findings demonstrate renal physiological protection by UA in a large-animal model of polymicrobial sepsis and support further evaluation of UA as an adjunctive intervention for SA-AKI.

## Introduction

Sepsis is a life-threatening syndrome caused by a dysregulated host response to infection that affects approximately 48.9 million people worldwide each year ^1^. It is the leading risk factor for acute kidney injury (AKI), with sepsis-associated AKI (SA-AKI) accounting for 26–50% of all AKI cases and occurring in up to 60% of patients with sepsis ^2,3^. The development of SA-AKI worsens clinical outcomes and is associated with substantially increased morbidity and mortality compared with sepsis alone ^2,4^. Beyond worsening the acute course of sepsis, SA-AKI predisposes survivors to chronic kidney disease and kidney failure requiring kidney replacement therapy ^2,4^. SA-AKI is diagnosed when AKI, defined according to KDIGO criteria by an increase in serum creatinine (≥ 0.3 mg/dL within 48 hours or ≥ 1.5-fold from baseline within 7 days) and/or oliguria (< 0.5 mL/kg/h for at least 6 hours), develops within 7 days of a confirmed or suspected diagnosis of sepsis ^5^. Current treatment of sepsis relies primarily on early antimicrobial therapy, fluid management, vasopressors, and supportive measures, including hemodynamic optimization and avoidance of nephrotoxic insults, which may reduce the risk of AKI development ^4^. However, despite advances in the clinical management of sepsis, there are currently no approved treatments that specifically prevent or treat SA-AKI ^4^. Therefore, identifying novel therapies capable of preserving renal function and targeting the mechanisms underlying SA-AKI remains an important unmet clinical priority.

Kidney dysfunction in SA-AKI results from a complex interplay of hemodynamic, inflammatory, immune, and metabolic disturbances ^5,6^. Its pathogenesis is multifactorial and is not yet fully understood, involving systemic and intrarenal inflammation, complement activation, oxidative stress, circulatory and microvascular dysfunction, tissue hypoxia, mitochondrial injury, cellular metabolic reprogramming, dysregulation of the renin–angiotensin–aldosterone system, and regulated forms of cell death ^5,6^. Among these mechanisms, increasing evidence has identified pyroptosis as a key contributor to the pathogenesis of sepsis and SA-AKI ^7^. Pyroptosis is a highly inflammatory form of programmed cell death that serves as an important host defense mechanism against infection but, when excessive or sustained, contributes to collateral tissue injury ^7,8^. In the canonical pathway, pyroptosis is initiated by the activation of inflammasomes by pathogen-associated molecular patterns (PAMPs) and damage-associated molecular patterns (DAMPs) generated during infection and tissue injury. The nucleotide-binding domain, leucine-rich repeat family, pyrin domain-containing 3 (NLRP3) inflammasome, a multiprotein complex composed of NLRP3, the adaptor protein ASC, and caspase-1, has been identified as a critical mediator of kidney injury in sepsis, with experimental studies demonstrating that its activation promotes tubular damage, inflammation, and renal dysfunction ^6,7,9,10^. Upon inflammasome activation, caspase-1 is activated and cleaves the pro-inflammatory cytokines pro-interleukin-1β (pro-IL-1β) and pro-interleukin-18 (pro-IL-18) into their mature forms, while also cleaving gasdermin D (GSDMD) ^8,9^. Cleavage of GSDMD is the pivotal event in pyroptosis, as its N-terminal fragment oligomerizes within the plasma membrane to form membrane pores, leading to cell swelling, membrane rupture, and the release of intracellular contents, including the mature cytokines IL-1β and IL-18, amplifying the inflammatory response ^8,9^. In the kidney, excessive activation of the NLRP3/caspase-1/GSDMD pathway promotes tubular epithelial cell injury, impaired renal function, and progression of SA-AKI ^7,8^, positioning pyroptosis as a potential therapeutic target for limiting kidney injury during sepsis.

Urolithins are bioactive gut microbiota-derived metabolites from ellagitannins and ellagic acid, which are dietary polyphenols present in pomegranates, walnuts, berries, and other plant-based foods^11^. Although ellagitannin-rich foods have long been associated with beneficial health effects, urolithins have gained recognition as one of the main drivers for these effects. Among these metabolites, urolithin A (UA) has attracted significant interest owing to its diverse biological activities^11^. UA has shown beneficial actions in numerous experimental models of health and disease. Preclinical studies have reported that UA exerts potent anti-inflammatory effects in animal models of intestinal inflammation, renal ischemia–reperfusion injury, osteoarthritis, peritonitis, sepsis-induced lung injury, and drug-induced nephrotoxicity ^11–15^. Beyond its anti-inflammatory actions, UA has also exhibited antioxidant, neuroprotective, cardiometabolic, anticancer, and mitochondria-protective properties ^14,16–18^. Early clinical studies have demonstrated that oral UA supplementation is safe and well tolerated and improves biomarkers of mitochondrial health, muscle function, and cardiovascular risk in healthy older adults^16,19^. Despite UA’s promising biological effects and favorable safety profile, its therapeutic potential has not been evaluated in SA-AKI.

Large-animal models provide an important translational bridge between mechanistic studies in rodents and clinical investigation in critically ill patients because they permit detailed assessment of systemic and organ-specific physiology using approaches relevant to human critical care. In the present study, we used a porcine model of sepsis induced by autologous fecal peritonitis to determine whether UA preserves renal function during evolving sepsis. We hypothesized that early administration of UA would attenuate sepsis-induced systemic and renal hemodynamic dysfunction and preserve kidney function. To test this hypothesis, we simultaneously assessed mean arterial pressure, cardiac output, renal blood flow, cortical and medullary perfusion, renal vascular resistance, and glomerular filtration rate, together with biochemical indices of kidney injury, inflammation, and oxidative stress. We also examined renal NLRP3 inflammasome-associated and downstream pyroptotic signaling and performed complementary studies in lipopolysaccharide-challenged primary porcine proximal tubular epithelial cells. This integrated large-animal approach was designed to characterize the physiological renal response to sepsis while evaluating UA as a candidate intervention for preserving kidney function during critical illness.

## Materials and Methods

### Animals

**Figure 1:**
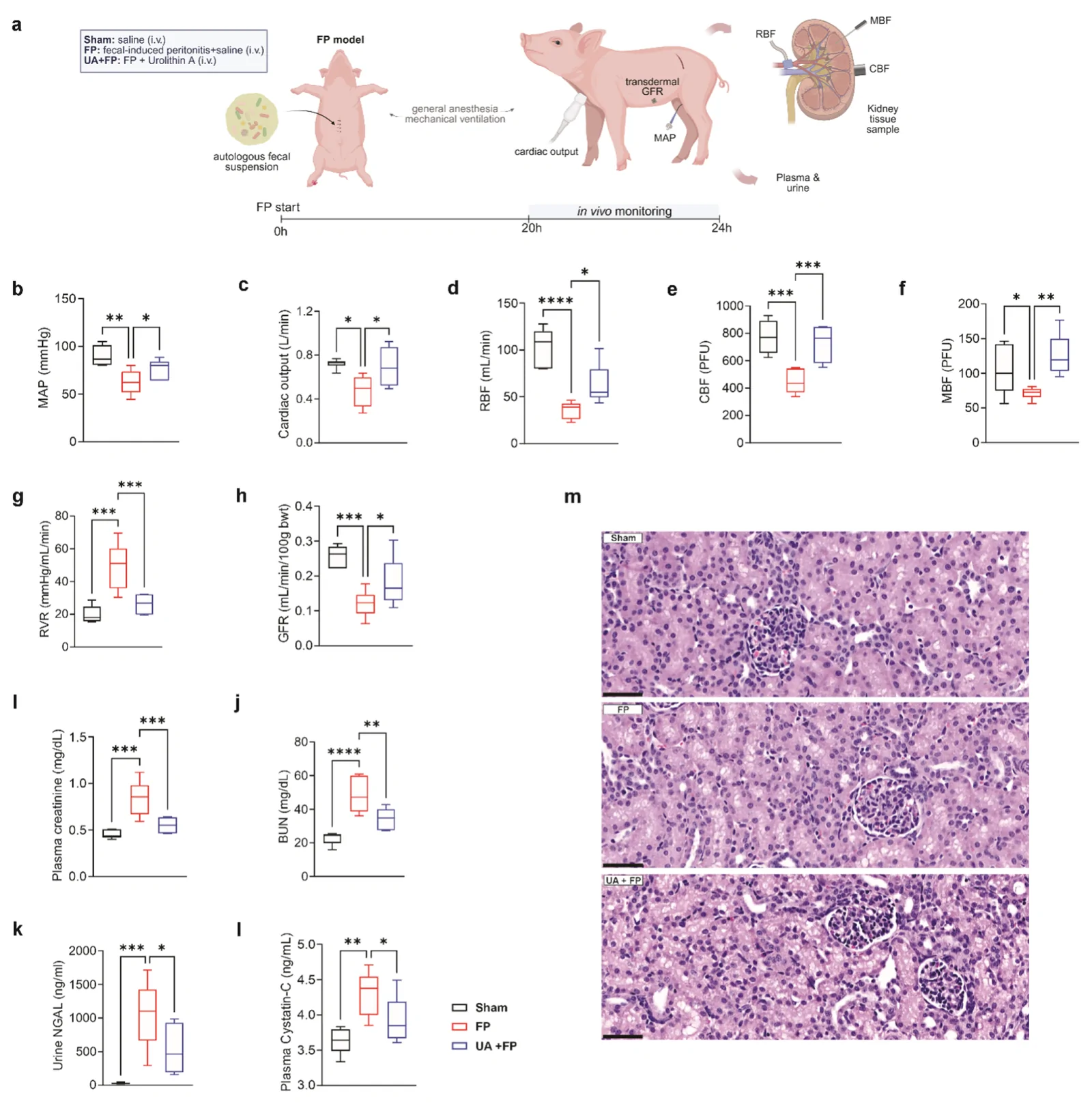
Urolithin A preserves systemic and renal hemodynamics and kidney function during fecal peritonitis-induced sepsis in pigs. **a**: Experimental design (Created with BioRender.com). Pigs were randomized to sham operation, fecal peritonitis (FP), or FP plus urolithin A (UA). Sepsis was induced by intraperitoneal administration of autologous fecal slurry, and UA was administered intravenously following sepsis induction. At 20 h, animals were re-anesthetized to assess systemic and renal hemodynamics and glomerular filtration rate (GFR) over 4 h, followed by blood and urine collection and kidney harvest. **b-m**: Mean arterial pressure (MAP; **b**), cardiac output (CO; **c**), renal blood flow (RBF; **d**), cortical blood flow/perfusion (CBF; **e**), medullary blood flow/perfusion (MBF; **f**), and renal vascular resistance (RVR; **g**). H-L: GFR (**h**), plasma creatinine (**i**), blood urea nitrogen (BUN; **j**), urinary neutrophil gelatinase-associated lipocalin (NGAL; **k**), and plasma cystatin C (**l**). **m**: Representative hematoxylin and eosin-stained kidney sections and renal injury scores. Data are presented as mean ± SEM. *P < 0.05, **P < 0.01, ***P < 0.001, and ****P < 0.0001, as indicated; one-way ANOVA followed by Holm-Sidak multiple-comparisons test; n = 6 pigs/group. UA, urolithin A.

All procedures followed national standards for the care and use of laboratory animals. The Animal Care and Use Committee (ACUC) of the University of Missouri, Columbia (MU), reviewed and approved the protocol (# 43406). This study is aligned with the MQTiPSS guidelines for improvement of animal modeling in sepsis research ^20^ and was designed, conducted, and reported in accordance with the Animal Research: Reporting of In Vivo Experiments (ARRIVE) guidelines 2.0 ^21^. All efforts were made to ensure the highest standards of animal welfare, experimental rigor, and transparency in reporting. Six- to eight-week-old male Landrace × Large White crossbred pigs (*Sus scrofa domesticus*) were obtained from MU Swine Farm (Columbia, MO). Following acclimatization and before surgery, pigs were randomly allocated to the experimental groups: 1) Sham with vehicle instillation (Sham); 2) Fecal peritonitis (FP)-induced sepsis (FP); 3) FP with UA (UA) administration (UA + FP). The sample size was determined based on previous studies using porcine models of sepsis conducted by our group ^22,23^. The animal experiments comprised two separate surgical procedures: one for fecal peritonitis induction and an end-of-study surgery for systemic and renal hemodynamics and glomerular filtration rate (GFR) monitoring (Fig. 1A).

### Fecal peritonitis induction

The pigs were premedicated with Zoletil^®^ 100 (tiletamine hydrochloride/zolazepam hydrochloride; Virbac, Carros, France) at a dose of 5 mg/kg body weight (BW) administered intramuscularly. Once sedated, the pigs were placed in a lateral position on a heating pad. The depth of anesthesia was confirmed before performing endotracheal intubation using a 5.0-mm endotracheal tube. Tube placement was assisted using a size-3 Miller blade laryngoscope. Anesthesia was induced and maintained with isoflurane (5–2%) delivered through a DRE Premier XP anesthesia machine connected to a DRE Puma V6 mechanical ventilator (DRE Veterinary, Louisville, KY, USA). Animals were mechanically ventilated throughout the procedure. Oxygen saturation (SpO_2_), heart rate, and end-tidal carbon dioxide (EtCO_2_) were continuously monitored using pulse oximetry and capnography, respectively. Vascular access was established by placement of a 24-gauge peripheral intravenous (IV) catheter (BD Insyte^™^ Autoguard^™^ Peripheral IV Catheter; BD, Franklin Lakes, NJ, USA) in either the saphenous or auricular vein. Maintenance fluid therapy was carried out using Lactate Ringer at 4 ml/kg BW/h. Immediately following induction of anesthesia, fresh fecal material was aseptically collected from each pig using a sterile rectal swab lubricated with a sterile, water-based lubricant. The autologous fecal slurry was prepared by suspending the collected feces (500 mg/kg BW) in 15 mL of sterile 0.9% saline (Baxter, Deerfield, IL, USA) and maintained at 38°C until administration ^24^.

The pigs were prepared for sterile surgery by clipping the abdominal hair and positioning them in dorsal recumbency on a surgical table. The surgical field was aseptically prepared using alternating scrubs of 70% ethanol and povidone–iodine (Betadine^®^, Avrio Health L.P., Stamford, CT, USA) and draped with a fenestrated sterile surgical drape. Adequate anesthetic depth was confirmed prior to performing a supra-umbilical midline minilaparotomy. An 18-Fr gastric tube was safely advanced into the peritoneal cavity. Intraperitoneal instillation of the fecal slurry was performed using a 60 mL syringe connected to the feeding tube (Feeding Tube Kit: 60 mL catheter syringe and 18-Fr feeding tube; LBH MARKET). After tube removal, the incision was closed in layers using 3 – 0 Vicryl^®^ suture (J393H; Ethicon, Johnson & Johnson, Somerville, NJ, USA). The peritoneal, muscular, and subcutaneous layers were approximated using continuous sutures, while the skin was closed with simple interrupted sutures. The pigs recovered from anesthesia on a heating pad while vital signs were monitored until they regained sternal recumbency. They were then returned to heated pens and housed individually for 20 hours. Analgesia was achieved in all animals by intramuscular injection of buprenorphine hydrochloride (0.3 mg/mL; Covetrus, Portland, ME, USA; 059122) at 0.01 mg/kg BW every 12 hours, initiated at the start of the surgical procedure. No antibiotics were administered during the study. Sham-operated pigs underwent the same surgical procedure as described above but received an intraperitoneal instillation of 15 mL of warm sterile 0.9% saline (Baxter, Deerfield, IL, USA) instead of fecal slurry.

The pigs from the UA + FP group were administered Urolithin A (UA; S5312; Selleck Chemicals LLC, Houston, TX, USA) intravenously at a dose of 5 mg/kg BW during the anesthetic recovery period, whereas the Sham and FP pigs received a bolus of Lactated Ringer’s solution (0.2 mL/kg BW). The dose was determined based on published studies ^25,26^. Consistent with the principle of reducing animal numbers in research, a Sham + UA-alone group was not included because existing evidence indicates that UA administration alone neither alters renal function nor induces kidney injury in healthy animals. ^27^. The time point for administration was 15 minutes after skin closure.

### Renal and systemic hemodynamics measurement

After a recovery period of 20 hours, the pigs were anesthetized using a mixture of 20 mg/kg of Ketamine (100 mg/ml, Covetrus, Dublin, OH, USA) and 2.2 mg/kg of Xylazine (100 mg/ml, Covetrus, Dublin, OH, USA), administered intramuscularly into the semimembranosus muscle of the rear leg. Subsequently, the piglets were intubated with a 5.0-mm cuffed endotracheal tube and positioned supine on a heating pad to maintain body temperature. Mechanical ventilation was provided using a pressure-controlled ventilator (Sechrist Millennium^®^, Sechrist Industries Inc., Anaheim, CA, USA). The femoral vein was catheterized for fluid administration and anesthetic induction and maintenance using α-chloralose intravenously, at a 50 mg/kg loading dose, followed by 20 mg/kg intermittent boluses. Arterial blood gas, pH, and hematocrit were monitored periodically throughout the experiment with a GEM Premier 5000 Blood Gas Analyzer (Instrumentation Laboratory, Bedford, MA). Ventilator settings were adjusted to maintain PCO_2_, PO_2_, and pH at physiological levels (~ 30 mm Hg, > 85 mm Hg, and 7.4, respectively). Animals were monitored repeatedly during experiments for anesthesia depth and re-dosed as needed. Cardiac output (CO) was measured non-invasively using a continuous-wave Doppler ultrasound cardiac output monitor (USCOM 1A^®^, USCOM Ltd., Sydney, NSW, Australia). Mean arterial pressure (MAP) was continuously monitored via a 3.5-Fr Mikro-Tip^®^ pressure catheter (SPR-524, Millar Instruments, Houston, TX, USA) inserted into the right femoral artery and connected to a data acquisition system (ADInstruments, Colorado Springs, CO, USA). The animals were then positioned in a right lateral semi-recumbent position, and the left kidney was exposed retroperitoneally through a flank incision to access the renal pedicle. The main renal artery was carefully dissected, and a 2-mm transit-time perivascular ultrasound flow probe (Transonic PS Series, Transonic Systems Inc., Ithaca, NY, USA) was placed around the artery and connected to a flowmeter (Transonic Systems Inc.) to measure total renal blood flow (RBF). Local cortical (CBF) and medullary (MBF) perfusion were assessed using laser Doppler flowmetry (PeriFlux System 5000, Perimed AB, Järfälla, Sweden). Urine was collected through a ureteral catheter. All physiological signals were acquired simultaneously using a PowerLab data acquisition system and LabChart Pro software (ADInstruments, Colorado Springs, CO, USA). Baseline (0 h) hemodynamic measurements were obtained after all monitoring devices had been placed and stabilized, and data were recorded continuously throughout the experimental period. The pigs were maintained under anesthesia and mechanical ventilation for 4 h following the procedure.

### Glomerular filtration rate determination

Glomerular filtration rate (GFR) was determined using the three-compartment model of fluorescein isothiocyanate (FITC)-sinistrin clearance, a method previously described and validated by our group in piglets subjected to cecal ligation and puncture (CLP) sepsis ^23,28^. This technique estimates GFR from the clearance kinetics of FITC-sinistrin measured using a transdermal fluorescence detector. While animals remained under anesthesia, an optical transdermal GFR monitoring device (MediBeacon GmbH, Mannheim, Germany) was prepared and secured to a previously shaved and cleaned area of the flank using an adhesive patch. After a 3–5 min stabilization period to establish baseline fluorescence, FITC-sinistrin solution (50 mg/mL) was administered intravenously as a rapid, smooth bolus at 20 mg/kg body weight. FITC-sinistrin clearance was continuously recorded for 4 h during systemic and renal hemodynamic monitoring. At the end of the recording period, the device was removed, and the data were analyzed using MediBeacon Studio 3 software according to the manufacturer’s instructions.

### Sample Collection

Systemic and renal hemodynamics and GFR were continuously monitored for 4 h, concluding a total experimental period of 24 h following fecal peritonitis induction. Blood samples were then collected via the femoral venous catheter into heparinized tubes. Samples were centrifuged, and plasma was separated and stored at − 80°C until analysis. Urine samples were collected, centrifuged, and stored at − 80°C for subsequent analysis. Animals were euthanized with Euthasol^®^ (0.2 mL/kg; 20% sodium pentobarbital and phenytoin sodium), and kidneys were harvested, sectioned transversely, and either fixed in 4% buffered formalin for histopathological evaluation or snap-frozen and stored at − 80°C for molecular analyses.

### Assessment of Renal Function, Kidney Injury, Inflammation, and Oxidative Stress

Kidney function was evaluated by measuring GFR, plasma creatinine concentration, blood urea nitrogen (BUN), urinary neutrophil gelatinase-associated lipocalin (NGAL), and plasma cystatin-C. Plasma creatinine concentrations were determined by liquid chromatography–tandem mass spectrometry (LC–MS/MS) at the UAB/UCSD O’Brien Core Center for Acute Kidney Injury Research at the University of Alabama at Birmingham. Plasma BUN levels were measured using a colorimetric assay (Urea Nitrogen Colorimetric Detection Kit, K024-H1; Arbor Assays, Ann Arbor, MI, USA). Urinary NGAL concentrations were determined using a porcine NGAL enzyme-linked immunosorbent assay (ELISA) kit (BioPorto Diagnostics, Hellerup, Denmark), and plasma cystatin-C concentrations were measured using a porcine cystatin-C ELISA kit (Immunotag^™^ Porcine Cys-C ELISA, IT6218; G-Biosciences, St. Louis, MO, USA).

Systemic inflammation and sepsis-associated responses were evaluated by measuring plasma procalcitonin, tumor necrosis factor-alpha (TNF-α), interleukin-1β (IL-1β), and lactate concentrations. Plasma procalcitonin levels were measured using a human procalcitonin ELISA kit (MBS2024352; MyBioSource, San Diego, CA, USA), plasma TNF-α using a porcine TNF-alpha Quantikine ELISA kit (PTA00; R&D Systems, Minneapolis, MN, USA), and plasma IL-1β using a porcine IL-1 beta/IL-1F2 Quantikine ELISA kit (PLB00B; R&D Systems, Minneapolis, MN, USA). Plasma lactate concentrations were determined using a bioluminescent assay (Lactate-Glo^™^ Assay, J5021; Promega, Madison, WI, USA). Plasma lipopolysaccharide (LPS) levels were measured using a General Lipopolysaccharides (LPS) ELISA kit (orb1946412, Biorbyt, Cambridge, United Kingdom). Multiple proinflammatory mediators in kidney homogenates were quantified using a porcine cytokine antibody array (Porcine Cytokine Array GS50, GSP-CAA-50-1; RayBiotech Life, Peachtree Corners, GA, USA).

Renal oxidative stress was assessed by measuring urinary isoprostane concentrations using an enzyme immunoassay (Urinary Isoprostane EIA Kit, EA85; Oxford Biomedical Research, Rochester Hills, MI, USA), whereas systemic antioxidant status was evaluated by measuring plasma catalase activity using a colorimetric assay (Catalase Colorimetric Activity Kit, K033-H1; Arbor Assays, Ann Arbor, MI, USA).

### Histopathology

Histopathological analysis was performed independently by iHisto Histopathology Support (Salem, MA, USA). Formalin-fixed kidney samples (4% neutral-buffered formalin) were processed, dehydrated through graded ethanol concentrations, embedded in paraffin, sectioned, and stained with hematoxylin and eosin (H&E) by the University of Missouri Veterinary Medical Diagnostic Laboratory (VMDL; Columbia, MO, USA). A board-certified pathologist at iHisto subsequently evaluated H&E-stained sections in a blinded manner to assess renal injury. Unstained sections were returned for subsequent immunofluorescence analyses. Histological evaluation included assessment of inflammatory cell infiltration, tubular cast formation, tubular epithelial cell vacuolization, glomerulonephritis, mesangial expansion, tubular necrosis, and glomerular diameter. Lesions were semi-quantitatively scored using a four-point scale: 0 = no changes, 1 = mild, 2 = moderate, and 3 = severe.

### Immunofluorescence

Paraffin-embedded kidney sections were subjected to antigen retrieval by immersing the slides in antigen retrieval buffer (R-Buffer A, Cat. No. 62706-10, Electron Microscopy Sciences, Hatfield, PA, USA) using a 2100 Retriever system (Aptum Biologics Ltd., Southampton, United Kingdom). Following antigen retrieval, sections were washed with phosphate-buffered saline (PBS) and permeabilized with 0.1% Triton X-100 for 15 min at room temperature. Samples were then incubated with blocking buffer (Animal-Free Blocker and Diluent, Ready-to-Use, Cat. No. SP-5035-100, Vector Laboratories, Newark, CA, USA) to minimize non-specific antibody binding and subsequently incubated overnight at 4°C with the appropriate primary antibodies at the indicated dilutions. The following day, sections were washed with PBS and incubated with the corresponding secondary antibodies for 1 h at room temperature. After final washes, slides were mounted with coverslips, and images were acquired from 3–5 randomly selected fields using a Leica DMi8 THUNDER Imager microscope (Leica Microsystems, Wetzlar, Germany).

Fluorescence intensity was quantified using Fiji (ImageJ, version 1.54i; National Institutes of Health, Bethesda, MD, USA) after channel separation and normalized to DAPI intensity. Three to five images per animal were analyzed, and the mean fluorescence intensity from all images was averaged to generate a single value per animal for statistical analysis.

### In vitro assessment of UA in LPS-challenged PTECs

Primary porcine renal proximal tubular epithelial cells (PTECs; Catalog #P-6015, Cell Biologics Inc., Chicago, IL, USA) were cultured in complete epithelial cell medium under standard conditions. Cells were seeded into 96-well and 6-well plates and assigned to one of four treatment groups: (1) Control (vehicle-treated), (2) LPS (10 μg/mL; lipopolysaccharide (LPS), S7850, Selleck Chemicals LLC, Houston, TX, USA), (3) Urolithin A (UA; 40 μM; S5312, Selleck Chemicals LLC, Houston, TX, USA), or (4) UA + LPS. For the UA + LPS group, cells were treated with UA in the presence of LPS. Following 24 h of incubation, cells cultured in 96-well plates were used to assess caspase-1 activity using the Caspase-Glo^®^ 1 Assay (G9951, Promega Corporation, Madison, WI, USA), whereas cells cultured in 6-well plates were harvested for Western blot analysis.

### Western immunoblotting

Total protein was extracted from primary porcine proximal tubular epithelial cells using RIPA lysis buffer. Protein concentrations were determined, and samples were denatured in LDS sample buffer containing dithiothreitol and heated at 70°C for 10 min. Proteins were separated by SDS-PAGE and transferred to PVDF membranes by semidry blotting. Membranes were blocked for approximately 1 h at room temperature in Tris-buffered saline containing 0.05% Tween-20 and 4% bovine serum albumin, then incubated overnight at 4°C with appropriate primary antibodies. After washing, membranes were incubated with horseradish peroxidase-conjugated secondary antibodies for approximately 1 h at room temperature. Immunoreactive bands were visualized using enhanced chemiluminescence. Where appropriate, membranes were cut into sections to allow incubation with different primary antibodies. Alternatively, membranes were stripped using Restore PLUS Western Blot Stripping Buffer (46430; Thermo Scientific, Rockford, IL, USA) and re-probed with additional primary antibodies.

### Antibodies

Mouse monoclonal anti-NLRP3 (1:200 for Western blotting and 1:100 for immunofluorescence; MA5-34969, Invitrogen). Rabbit monoclonal anti-cleaved caspase-1 (1:500 for Western blotting and 1:100 for immunofluorescence; Asp296, Cell Signaling Technology). Rat monoclonal anti-gasdermin D N-terminal fragment (1:300 for Western blotting and 1:100 for immunofluorescence; 939702, BioLegend). Rabbit polyclonal anti-IL-1β (1:100; P420B, Invitrogen). Mouse monoclonal anti-IL-18 (1:100; 60070-1-Ig, Proteintech). Mouse monoclonal anti-4-hydroxynonenal (1:100; MA2-7570, Invitrogen). Rabbit polyclonal anti-nitrotyrosine (1:100; ab42789, Abcam). Rabbit polyclonal anti-TMS1/ASC (1:100; ab309497, Abcam).

Rabbit Anti-Mouse IgG H&L (HRP) (1/5000; ab6728, abcam). Anti-rabbit IgG, HRP-linked Antibody (1/5000; 7074, Cell Signaling Technology). Goat Anti-Rat IgG H&L (HRP) (1/5000; ab205720, abcam). CF555 Donkey anti-rabbit IgG (1/400; 20038-1, Biotium Inc, Fremont, CA). CF488A Goat anti-mouse IgG (1/400; 20018-1, Biotium Inc, Fremont, CA). Goat Anti-Rat IgG H&L (Alexa Fluor^®^ 488) (1/400; ab150157, abcam).

### Statistical analysis

Statistical analyses and graphing were performed using GraphPad Prism version 11.0.2 (GraphPad Software, Sacramento, CA, USA). Data are presented as mean ± SEM and as box-and-whisker plots showing the minimum and maximum values. Renal hemodynamic variables were normalized to kidney weight, and renal vascular resistance was calculated as mean arterial pressure divided by renal blood flow normalized to kidney weight. Group comparisons were performed using one-way ANOVA followed by Holm-Sidak post hoc testing. Kidney cytokine-array data were normalized by row as Z scores and displayed as a heatmap. Statistical significance was defined as P < 0.05.

## Results

### UA preserved systemic and renal hemodynamics and kidney function after fecal peritonitis

At 24 h after fecal peritonitis, FP pigs exhibited systemic hemodynamic impairment, with mean arterial pressure reduced by more than 30% and cardiac output reduced by 34% compared with sham animals (Fig. 1B–C). UA-treated FP pigs had higher mean arterial pressure and cardiac output than untreated FP pigs. FP also impaired renal circulation, as indicated by lower renal blood flow, cortical perfusion, and medullary perfusion, together with increased renal vascular resistance (Fig. 1D–G). UA attenuated each of these abnormalities. Consistent with the hemodynamic changes, FP reduced GFR and increased plasma creatinine, blood urea nitrogen, urinary NGAL, and plasma cystatin C (Fig. 1H–L). UA preserved GFR and reduced these biochemical indices of kidney dysfunction. Histological changes at this early time point were mild and consisted predominantly of tubular vacuolation; no injury score exceeded 2 (Fig. 1M). Inflammatory cell infiltration, tubular casts, glomerulonephritis or mesangial expansion, and tubular necrosis were not observed, and glomerular diameter did not differ among groups (83–109 μm).

### UA attenuated systemic and renal inflammatory responses

**Figure 2:**
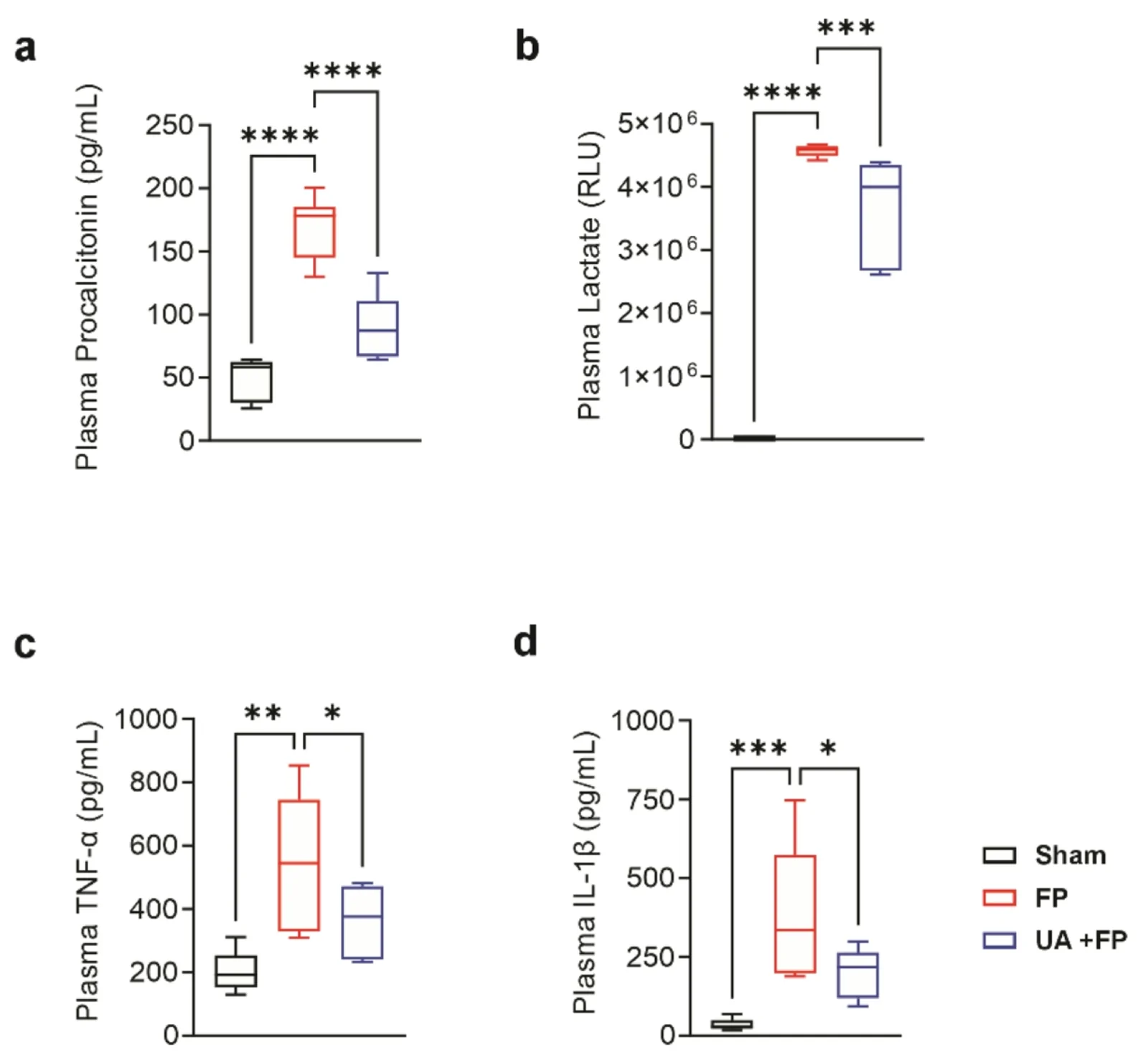
Urolithin A attenuates systemic inflammatory responses during fecal peritonitis-induced sepsis. Plasma concentrations of procalcitonin (**a**), lactate (**b**), tumor necrosis factor-α (TNF-α; **c**), and interleukin-1β (IL-1β; **d**) in sham-operated pigs, pigs subjected to fecal peritonitis (FP), and pigs subjected to FP and treated with urolithin A (UA). FP increased all four indices of systemic sepsis and inflammation, whereas UA attenuated these responses. Data are presented as mean ± SEM. *P < 0.05, **P < 0.01, ***P < 0.001, and ****P < 0.0001, as indicated; one-way ANOVA followed by Holm-Sidak multiple-comparisons test; n = 6 pigs/group. RLU, relative light units.

**Figure 3:**
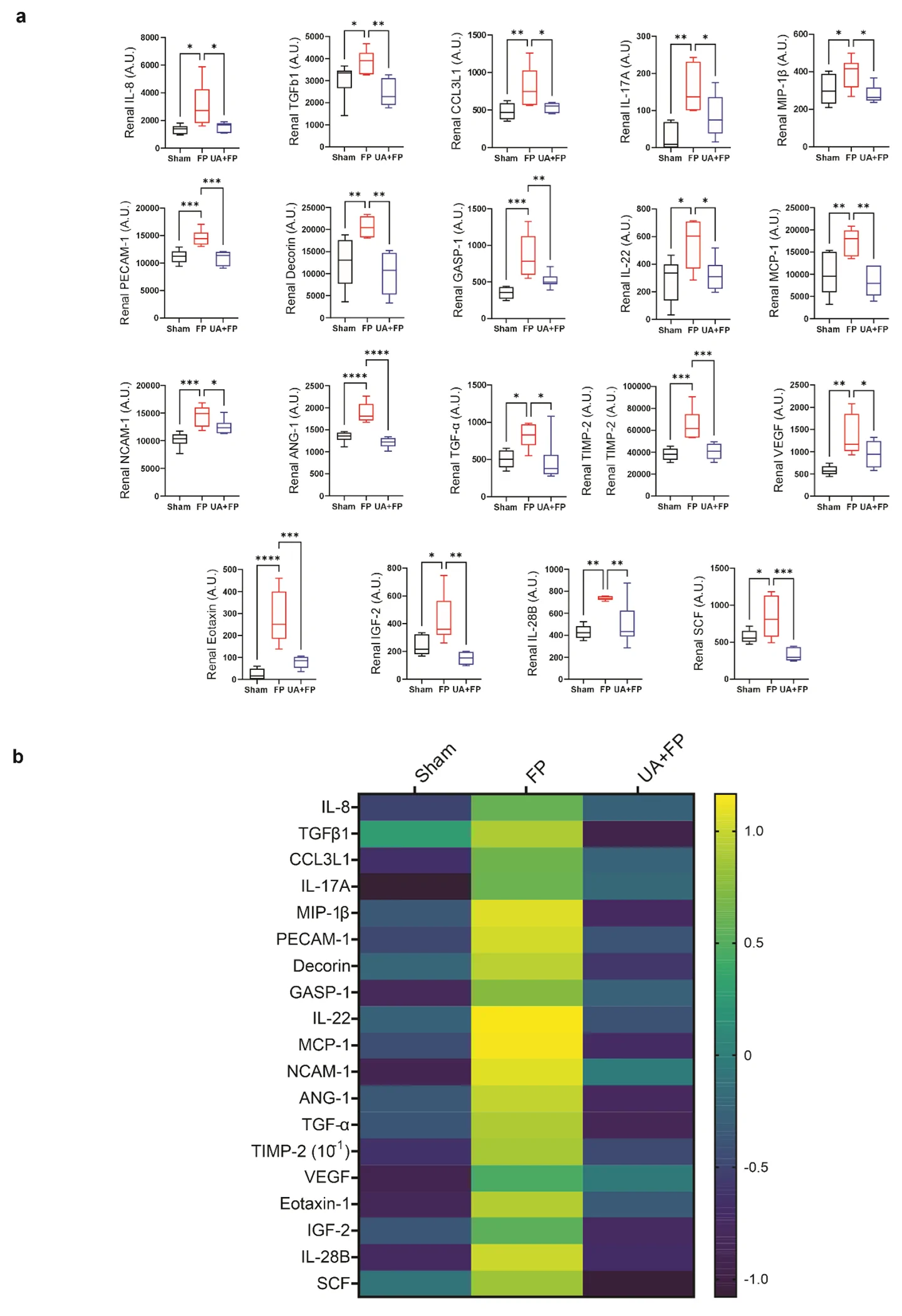
Urolithin A attenuates fecal peritonitis-induced changes in renal inflammatory mediators. Renal inflammatory mediators were measured in kidney lysates using a porcine cytokine antibody array. **a**: Quantification of mediators that differed significantly among sham, fecal peritonitis (FP), and FP plus urolithin A (UA) groups, expressed as arbitrary units. **b**: Heatmap of the corresponding renal mediators after row-wise Z-score normalization. FP produced a broad inflammatory response in the kidney that was attenuated by UA treatment. Data are presented as mean ± SEM. *P < 0.05, **P < 0.01, ***P < 0.001, and ****P < 0.0001, as indicated; one-way ANOVA followed by Holm-Sidak multiple-comparisons test; n = 6 pigs/group. ANG-1, angiopoietin-1; CCL3L1, C-C motif chemokine ligand 3-like 1; GASP-1, G protein-coupled receptor-associated sorting protein 1; IGF-2, insulin-like growth factor 2; IL, interleukin; MCP-1, monocyte chemoattractant protein-1; MIP-1β, macrophage inflammatory protein-1β; NCAM-1, neural cell adhesion molecule-1; PECAM-1, platelet endothelial cell adhesion molecule-1; SCF, stem cell factor; TGF, transforming growth factor; TIMP-2, tissue inhibitor of metalloproteinases-2; VEGF, vascular endothelial growth factor.

FP increased plasma procalcitonin and lactate, consistent with the development of systemic sepsis ^29,30^ (Fig. 2A–B). Plasma TNF-α and IL-1β were also increased (Fig. 2C–D). UA treatment reduced procalcitonin, lactate, TNF-α, and IL-1β compared with untreated FP animals. In kidney lysates, FP increased multiple inflammatory mediators measured with a porcine cytokine array (Fig. 3A). Z-score-normalized heatmap analysis showed a broad renal inflammatory response in FP animals, whereas the profile in UA-treated FP kidneys shifted toward that of sham animals (Fig. 3B).

### UA reduced renal and systemic oxidative stress

**Figure 4:**
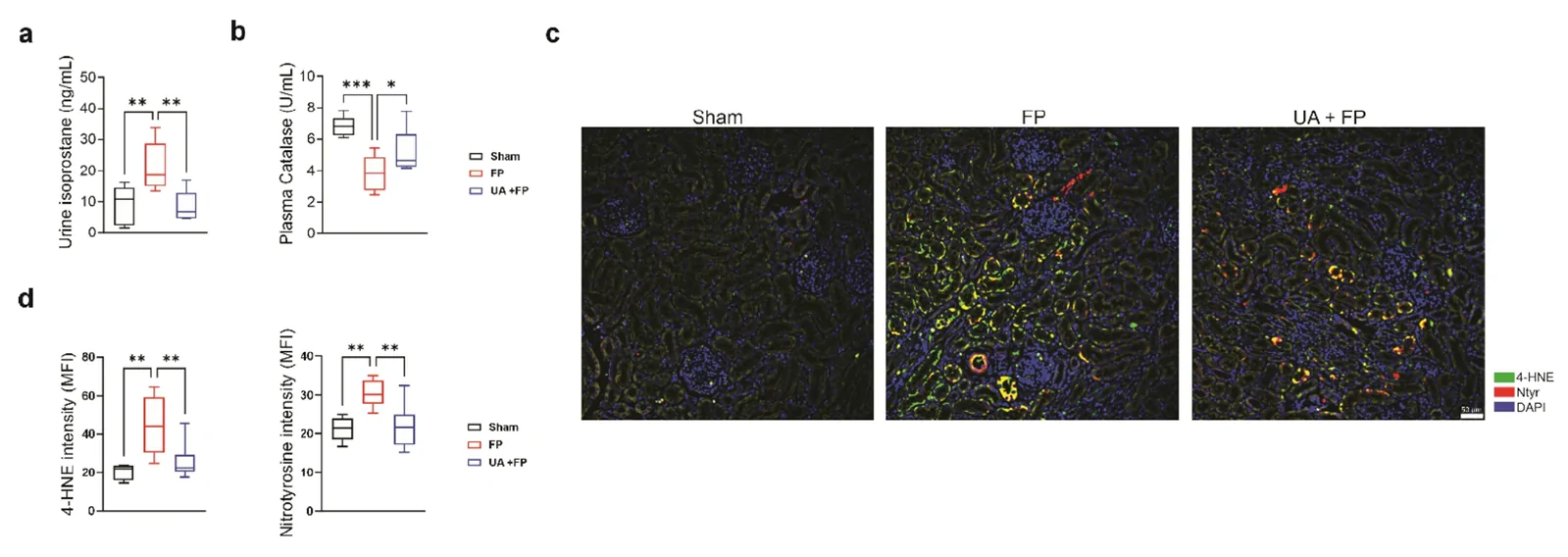
Urolithin A reduces systemic and renal oxidative stress during fecal peritonitis-induced sepsis. **a**: Urinary isoprostane concentration. **b**: Plasma catalase activity. **c-d**: Representative immunofluorescence images and quantitative fluorescence intensity of renal nitrotyrosine and 4-hydroxynonenal (4-HNE) staining in sham-operated pigs, pigs subjected to fecal peritonitis (FP), and pigs subjected to FP and treated with urolithin A (UA). Nuclei were counterstained with DAPI. FP increased urinary isoprostane and renal nitrotyrosine and 4-HNE immunoreactivity and reduced plasma catalase activity; these alterations were attenuated by UA. Data are presented as mean ± SEM. *P < 0.05, **P < 0.01, and ***P < 0.001, as indicated; one-way ANOVA followed by Holm-Sidak multiple-comparisons test; n = 6 pigs/group. MFI, mean fluorescence intensity. Scale bar = 50 μm.

FP increased urinary isoprostane and reduced plasma catalase activity, indicating increased renal oxidative stress and impaired antioxidant defense (Fig. 4A–B). UA lowered urinary isoprostane and preserved plasma catalase activity. In kidney sections, FP increased immunoreactivity for 4-hydroxynonenal, a product of lipid peroxidation, and nitrotyrosine, a marker of nitrosative stress. Both signals were reduced in UA-treated FP kidneys (Fig. 4C–D).

### UA attenuated renal NLRP3 inflammasome-associated and pyroptotic signaling

**Figure 5:**
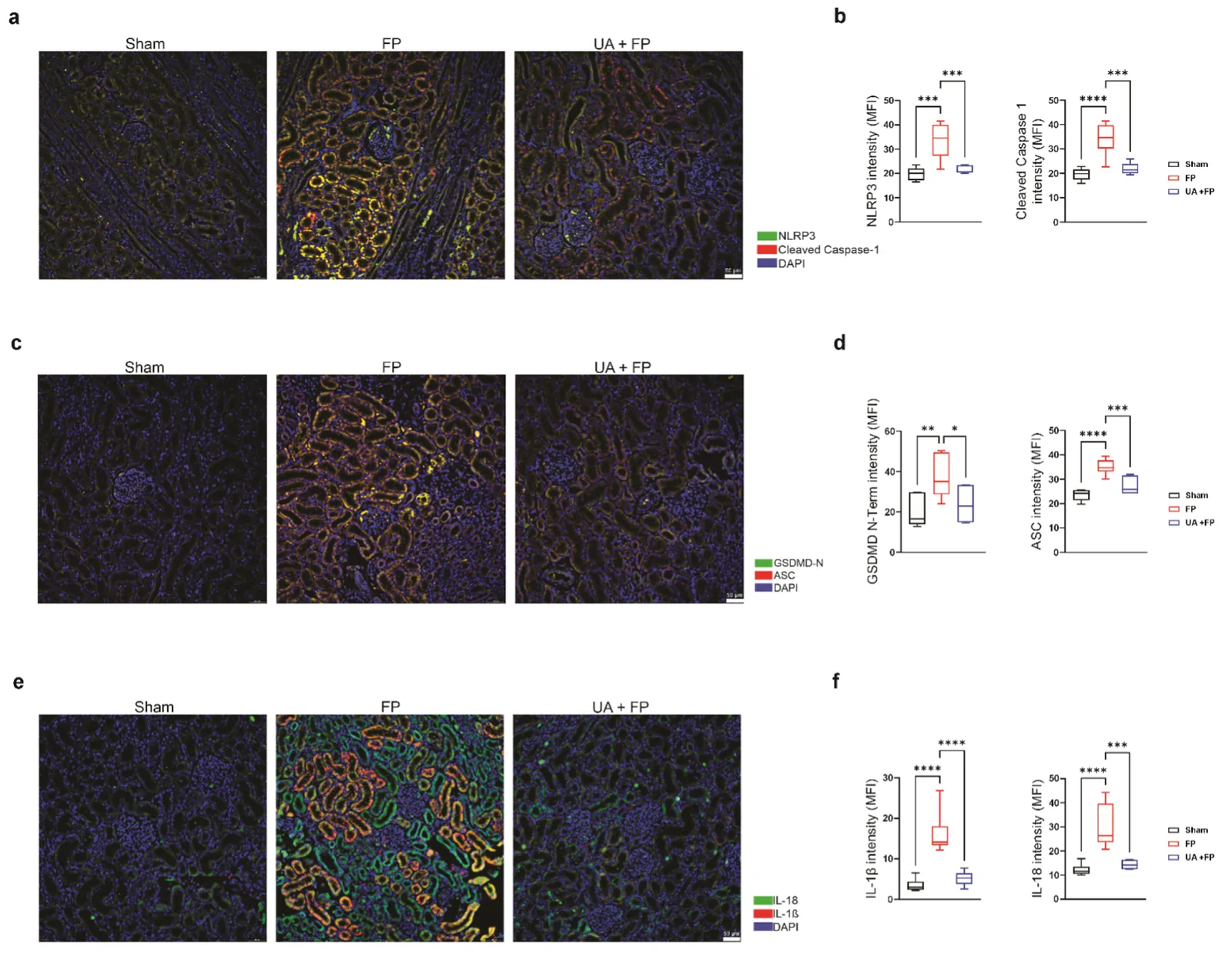
Urolithin A attenuates renal NLRP3 inflammasome-associated and pyroptotic signaling during fecal peritonitis-induced sepsis. Representative immunofluorescence images and quantitative fluorescence intensity of inflammasome- and pyroptosis-associated proteins in kidneys from sham-operated pigs, pigs subjected to fecal peritonitis (FP), and pigs subjected to FP and treated with urolithin A (UA). **a-b**: NLR family pyrin domain containing 3 (NLRP3) and cleaved caspase-1. **c-d**: Gasdermin D N-terminal fragment (GSDMD-NT) and apoptosis-associated speck-like protein containing a caspase recruitment domain (ASC). **e-f**: Interleukin-1β (IL-1β) and interleukin-18 (IL-18). FP increased renal immunoreactivity for each marker, whereas UA treatment attenuated these responses. Nuclei were counterstained with DAPI. Data are presented as mean ± SEM. *P < 0.05, **P < 0.01, ***P < 0.001, and ****P < 0.0001, as indicated; one-way ANOVA followed by Holm-Sidak multiple-comparisons test; n = 6 pigs/group. MFI, mean fluorescence intensity. Scale bar = 50 μm.

Kidney sections were immunostained for proteins associated with canonical NLRP3 inflammasome activation and downstream pyroptotic signaling. FP increased renal immunoreactivity for NLRP3, ASC, and cleaved caspase-1, as well as the gasdermin D N-terminal fragment, IL-1β, and IL-18 (Fig. 5). UA-treated FP kidneys showed lower immunoreactivity for each marker than untreated FP kidneys. These findings indicate that the functional protection associated with UA was accompanied by attenuation of renal inflammasome-associated pyroptotic signaling.

### Urolithin A attenuated LPS-induced inflammasome-associated responses in porcine proximal tubular cells

**Figure 6:**
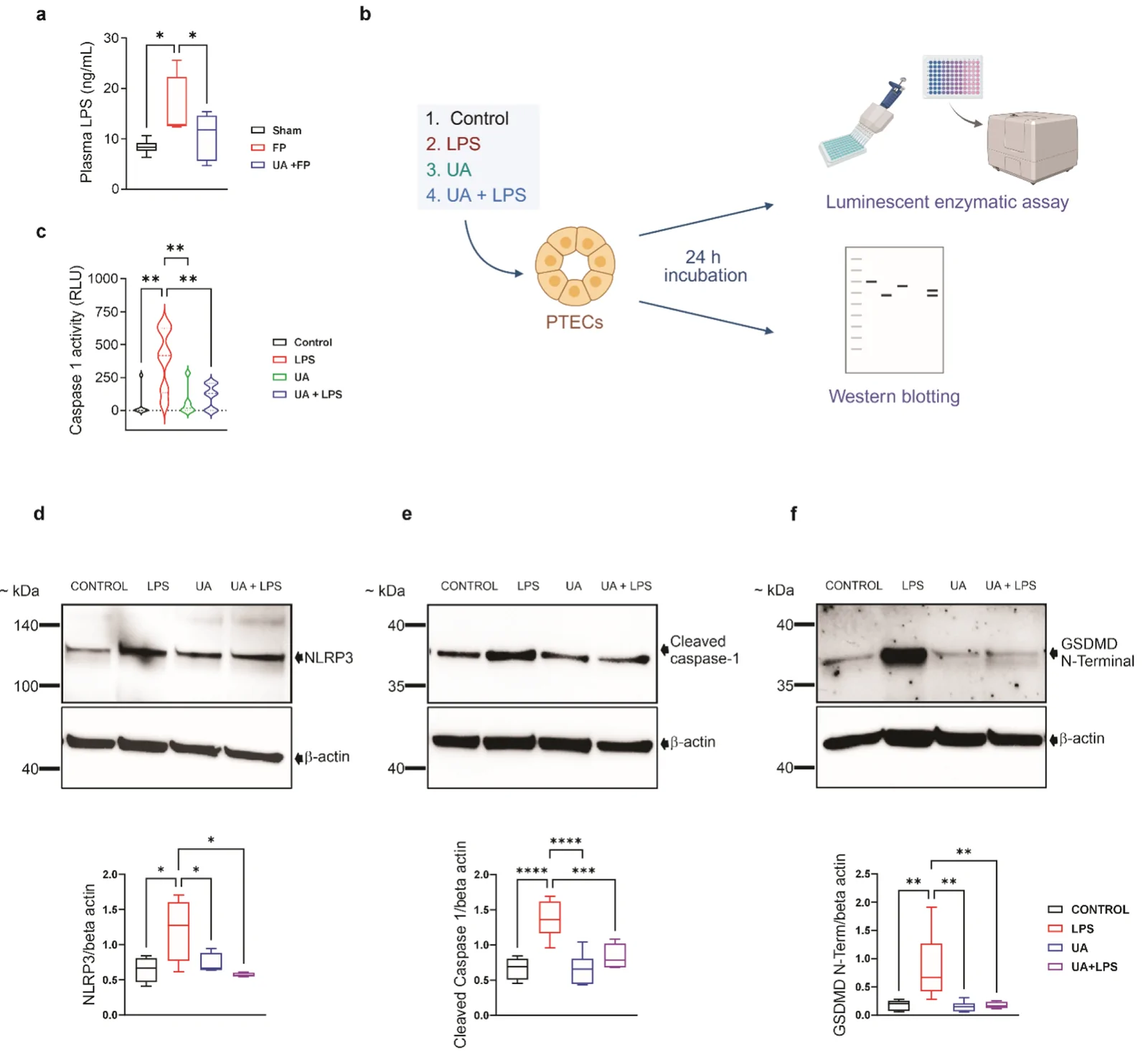
Urolithin A attenuates LPS-induced inflammasome and pyroptotic signaling in primary porcine proximal tubular epithelial cells. **a**: Plasma lipopolysaccharide (LPS) concentrations in sham-operated pigs, pigs subjected to fecal peritonitis (FP), and pigs subjected to FP and treated with urolithin A (UA). **b**: Experimental design of the in vitro studies. Primary porcine proximal tubular epithelial cells (PTECs) were treated with vehicle, LPS, UA, or UA plus LPS for 24 h before assessment of caspase-1 activity and protein expression (Created with BioRender.com). **c**: Caspase-1 activity measured by luminescent assay. **d-f**: Representative immunoblots and densitometric analysis of NLR family pyrin domain containing 3 (NLRP3; **d**), cleaved caspase-1 (**e**), and gasdermin D N-terminal fragment (GSDMD-NT; **f**). β-Actin was used as the loading control. UA attenuated LPS-induced caspase-1 activation and expression of NLRP3, cleaved caspase-1, and GSDMD-NT. Membranes were either sectioned for incubation with different primary antibodies or stripped and re-probed with additional primary antibodies. Data are presented as mean ± SEM. *P < 0.05, **P < 0.01, ***P < 0.001, and ****P < 0.0001, as indicated; one-way ANOVA followed by Holm-Sidak multiple-comparisons test; n = 6 pigs/group. FP, fecal peritonitis; RLU, relative light units.

Immunofluorescence signals for the evaluated inflammasome-associated proteins were observed predominantly in renal tubular regions. To examine a direct tubular-cell response, primary porcine proximal tubular epithelial cells were challenged with LPS, which was also elevated in the plasma of FP pigs (Fig. 6A). After 24 h of exposure (Fig. 6B), UA reduced LPS-induced caspase-1 activity (Fig. 6C). LPS also increased NLRP3, cleaved caspase-1, and gasdermin D N-terminal fragment protein abundance, whereas co-treatment with UA attenuated these responses (Fig. 6D–F).

## Discussion

In this study, we used a porcine model of autologous fecal peritonitis to examine renal physiological dysfunction during sepsis and to evaluate the effects of early UA administration on systemic and kidney function. Fecal peritonitis produced a hypodynamic septic phenotype characterized by systemic inflammation, reduced mean arterial pressure and cardiac output, impaired renal macrovascular and microvascular perfusion, increased renal vascular resistance, and decreased GFR. These physiological abnormalities were accompanied by increased biochemical indices of kidney injury, renal inflammation, oxidative stress, and enhanced NLRP3 inflammasome-associated and downstream pyroptotic signaling. UA administration attenuated systemic hemodynamic deterioration, preserved renal blood flow and cortical and medullary perfusion, reduced renal vascular resistance, and maintained GFR. These functional effects were accompanied by lower circulating and urinary kidney injury biomarkers and reduced inflammatory, oxidative, and inflammasome-associated responses. Complementary experiments in primary porcine proximal tubular epithelial cells further demonstrated that UA attenuated LPS-induced caspase-1 activity and expression of NLRP3, cleaved caspase-1, and the N-terminal fragment of gasdermin D. Together, these findings demonstrate that early UA administration preserves renal physiology during experimental porcine sepsis and identify suppression of inflammatory, oxidative, and inflammasome-associated signaling as potential contributors to this protection.

The hemodynamic profile observed 24 h after FP induction resembles the hypodynamic phase of severe sepsis, in which declining cardiac output and arterial pressure compromise organ perfusion ^31,32^. Other porcine FP studies have reported hyperdynamic circulation, likely reflecting differences in disease stage, model severity, and resuscitation ^24,33^. Park et al. demonstrated a time-dependent transition from an early hyperdynamic state to later cardiovascular depression in pigs subjected to FP ^34^. In the present study, UA increased mean arterial pressure and cardiac output and preserved renal blood flow and cortical and medullary perfusion. These measurements are a major strength because they show that GFR was preserved in the setting of improved renal macrovascular and microvascular perfusion. However, because UA also improved systemic hemodynamics, the current study cannot determine whether the renal benefit resulted from direct kidney action, attenuation of overall sepsis severity, or both. Fluid and vasopressor therapy remains central to the treatment of septic shock ^4^, and renal microcirculatory impairment may persist despite correction of systemic pressure ^24,35^. Future work will therefore be needed to determine whether UA provides additional benefit when combined with contemporary resuscitation strategies.

FP caused a clear decline in kidney function, reflected by reduced GFR and increased plasma creatinine, BUN, cystatin C, and urinary NGAL. UA improved each of these outcomes. These findings extend previous reports of UA-mediated renal protection in rodent models of ischemia-reperfusion injury and cisplatin nephrotoxicity ^13,14^ to a porcine model of polymicrobial sepsis. UA also reduced plasma TNF-α and IL-1β and shifted the renal cytokine profile toward sham levels, consistent with its reported anti-inflammatory actions ^13,17^. The present results do not identify a single upstream anti-inflammatory pathway, and mechanisms such as NF-κB modulation were not directly evaluated. Accordingly, the cytokine findings are best interpreted as evidence that UA attenuated the systemic and intrarenal inflammatory milieu accompanying physiological protection.

Oxidative stress is an important component of SA-AKI ^36,37^. FP increased urinary isoprostane, reduced plasma catalase activity, and increased renal 4-hydroxynonenal and nitrotyrosine immunoreactivity. UA attenuated each response, supporting antioxidant and cytoprotective effects in this model. Prior studies have linked UA to endogenous antioxidant pathways, mitochondrial quality control, and mitophagy ^12,14,17,18,38^, but these pathways were not measured here and therefore remain possible. Despite marked functional, inflammatory, and oxidative abnormalities, renal histological changes were mild at 24 h. This apparent dissociation is consistent with experimental and human observations that severe renal dysfunction during sepsis may occur with limited structural injury ^39,40^. The findings emphasize the value of direct physiological measurements, including renal perfusion and GFR, for characterizing early SA-AKI.

FP increased renal immunoreactivity for NLRP3, ASC, cleaved caspase-1, gasdermin D N-terminal fragment, IL-1β, and IL-18. These proteins are components or downstream products of the canonical inflammasome and pyroptotic signaling ^7–9^. UA reduced each signal, and the cell studies provided complementary evidence that UA attenuated LPS-induced caspase-1 activity and expression of NLRP3, cleaved caspase-1, and gasdermin D N-terminal fragment in porcine proximal tubular epithelial cells. These convergent observations support an association between UA treatment and reduced inflammasome-related signaling. However, they do not establish that pyroptotic cell death occurred in vivo or that inhibition of this pathway was causally responsible for the improvement in renal function. Because reliable Western immunoblot detection of these proteins could not be achieved in porcine kidney lysates with the available antibodies, the in vivo evidence is based on quantitative tissue immunofluorescence. Accordingly, the mechanistic interpretation should remain limited to attenuation of inflammasome-associated pyroptotic signaling rather than direct demonstration of pyroptosis.

Other limitations of this study include the use of a single intravenous dose of UA administered shortly after induction of fecal peritonitis and before established renal dysfunction was documented. Thus, the study primarily models early intervention rather than treatment of established SA-AKI. In addition, the pigs did not receive antibiotics, vasopressors, or protocolized resuscitation beyond maintenance fluids. Only young male pigs and a single 24-hour endpoint were studied, which further limits assessment of sex-, age-, and time-dependent responses. Moreover, because UA improved systemic inflammatory and hemodynamic abnormalities, the extent to which its renal benefits reflect direct kidney-specific effects versus secondary protection from improved systemic physiology cannot be determined. Despite these limitations, the translational relevance of UA is supported by its reported safety and biological activity in early human studies ^16,19^, and the present findings provide a rationale for future studies evaluating delayed treatment, standard-of-care sepsis therapy, and longer-term outcomes.

In conclusion, autologous fecal peritonitis in pigs produced systemic circulatory dysfunction accompanied by renal macrovascular and microvascular hypoperfusion, reduced GFR, biochemical evidence of AKI, oxidative stress, inflammation, and increased inflammasome-associated signaling. Early administration of UA preserved systemic and renal hemodynamics, maintained GFR, and reduced biochemical and molecular indices of kidney injury. These findings establish the feasibility of using a porcine polymicrobial sepsis model to integrate clinically relevant physiological measurements with molecular and cellular endpoints during preclinical therapeutic evaluation. Although additional studies are required to determine the efficacy of delayed UA administration alongside contemporary sepsis care and to establish the mechanisms responsible for renal protection, the present results support further investigation of UA as an adjunctive strategy for preserving kidney function during sepsis.

## Supplementary Material

This is a list of supplementary files associated with this preprint. Click to download.

- SupplementaryRepBlotsPTECs.pdf
- floatimage7.png

## Data Availability

The data and methods from this study are available from the corresponding author upon reasonable request.
